# Mitochondrial DNA Damage and Diseases

**DOI:** 10.12688/f1000research.6665.1

**Published:** 2015-07-01

**Authors:** Gyanesh Singh, U C Pachouri, Devika Chanu Khaidem, Aman Kundu, Chirag Chopra, Pushplata Singh

**Affiliations:** 1 School of Biotechnology and Biosciences, Lovely Professional University, Phagwara, Punjab, India; 2 Department of Medicine, Punjab Institute of Medical Sciences, Jalandhar, Punjab, India

**Keywords:** Oxidative damage, mitochondrial DNA repair, mitochondrial pathology, mitochondrial mutations

## Abstract

Various endogenous and environmental factors can cause mitochondrial DNA (mtDNA) damage.  One of the reasons for enhanced mtDNA damage could be its proximity to the source of oxidants, and lack of histone-like protective proteins. Moreover, mitochondria contain inadequate DNA repair pathways, and, diminished DNA repair capacity may be one of the factors responsible for high mutation frequency of the mtDNA. mtDNA damage might cause impaired mitochondrial function, and, unrepaired mtDNA damage has been frequently linked with several diseases. Exploration of mitochondrial perspective of diseases might lead to a better understanding of several diseases, and will certainly open new avenues for detection, cure, and prevention of ailments.

## Introduction

**Figure 1.**
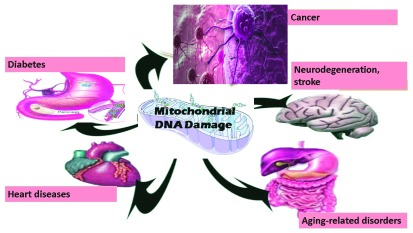
Mitochondrial DNA damage is associated with various diseases.

Mitochondria, a key organelle of most eukaryotic cells, are not only essential for cellular energy generation but also important for calcium metabolism and apoptotic cell-signaling ^1^. Like the nucleus, both mitochondria and chloroplasts contain their own DNA, and mitochondrial DNA (mtDNA) damage has been frequently implicated in several diseases including neurodegeneration, cancer, stroke, cardiomyopathy, diabetes, and aging-related disorders ( 2, Figure 1). Unlike nuclear DNA, the mitochondrial genome is circular, contains very few introns, and the number of mtDNA copies in one mitochondrion can be in the range of two to ten. Furthermore, the size of mtDNA is very small (16.6 kb in humans), and mitochondrial codon-usage is also different. The multicopy nature of mtDNA bestows unconventional modes of DNA maintenance such as selective degradation of damaged DNA, and an unusual form of recombination ^3^. mtDNA is maternally inherited, and sperm mitochondria are mostly degraded after fertilization ^4^. Mitochondria synthesize some of its own proteins, and one of the reasons for this could be that all proteins that are translated in cytoplasm might not be able to cross mitochondrial membranes owing to their varied hydrophobicity ^5^. mtDNA encodes 22 tRNAs, 2 rRNAs, and 13 proteins that participate in mitochondrial ATP synthesis ^6^. Reactive oxygen species (ROS) are very reactive oxygen-containing molecules. ROS are produced in all types of cells and can have various harmful effects. mtDNA, like other DNA, can not only be damaged by radiation and genotoxic chemicals but also by ROS that are frequently produced in mitochondria ^7^. mtDNA damage can exaggerate further because of errors during DNA replication, and lack of conventional histone proteins in mitochondria ^8^. ROS can cause various types of oxidative damage including DNA strand breaks, base modification or removal, and cross linking. DNA polymerase γ (pol γ), the only DNA polymerase known to be present in the mitochondria, have low frameshift fidelity, and, is believed to be a major contributor to changes in mtDNA ^9^.

## Consequences of mitochondrial DNA damage

Several studies report the effect of genotoxic agents on mitochondria ^10, 11^. However, it is not easy to draw conclusions in these cases, as agents that damage mtDNA also damage nuclear DNA. Therefore, it is suggested that all studies should compare consequences of nuclear and mtDNA damage in such cases, as far as possible. Other than its involvement in cancer and neurological disorders, changes in mtDNA have been shown to be associated with a few hereditary diseases ^12^. mtDNA damage is well known to cause impaired bioenergetics, reduced cell proliferation and apoptosis, hypercholesterolemia, and atherosclerosis ^12^. Interestingly, mtDNA defects are known to cause defective mitochondrial ATP generation that results into compromised organ function and diseases ^13^.

In case of the most common neurodegenerative disorders including Parkinson's disease (PD), Alzheimer's disease (AD), and amyotrophic lateral sclerosis (ALS) also, mtDNA damage has been implicated as a factor that cause or exaggerate these diseases ^14^. Brain tissues from Alzheimer's patients show greater fragmentation mtDNA. However, similar damage to nuclear DNA is controversial in this case. Increased mtDNA damage was also associated with reduced levels of mitochondrial protein expression ^13^. Interestingly, brain tissues from Alzheimer's patients show higher levels of oxidized bases. In this case, mtDNA was found to have 10-times more oxidized bases compared to nuclear DNA indicating that mtDNA is more succeptible to oxidants ^14^.

In the case of Huntington disease (HD), higher levels of oxidative stress were observed in the brain tissues of both humans and mice ^16^. In the case of a mouse model of HD, embryonic fibroblasts showed increased mitochondrial matrix Ca2+ loading, and higher superoxide generation. This confirmed that both mitochondrial Ca2+ signaling and superoxide generation are dysregulated in HD, and, reducing mitochondrial Ca2+ uptake can be a therapeutic strategy for HD ^16^. Peripheral blood mononuclear cells (PBMCs) from systemic lupus erythematosus (SLE) patients also exhibited enhanced mtDNA damage indicating potential role of mitochondria in the pathogenesis of SLE ^17^. Apolipoprotein E (ApoE) is known to play a protective role in preventing artery wall thickening in atherosclerosis and ApoE-/- mice show mtDNA damage before significant atherosclerosis ^18^. pol γ-/-/ApoE-/- mice show extensive mtDNA damage, impaired mitochondrial respiration, and increased atherosclerosis, even without increased ROS. Furthermore, pol γ-/-/ApoE-/- monocytes showed increased inflammatory cytokine release ^18^. Aging is often associated with the accumulation of deleterious changes, reduced physiological functions, and increased likelihood of diseases ^19^. In this context, a number of mitochondrial aberrations have been observed with aging. These aberrations are accumulation of mtDNA mutation, inefficient oxidative phosphorylation, increased production of ROS, and disorganized mitochondrial structure ^20^. These mtDNA mutations are often somatic, with variable changes in individual cells. Often, higher levels of these mutations are associated with respiratory chain deficiency. A mosaic pattern of respiratory chain deficiency can be found in different tissues because of uneven distribution of mutations ^13^. The mitochondrial free radical theory of aging has been one of the most supported ideas of aging ^19^. This theory postulates that the production of intracellular ROS is the major determinant during aging. Several invertebrate and mammalian models already support this hypothesis. Oxidative stress, when propagated by active radicals, can damage DNA, phospholipids, proteins and other biomolecules. Reactive oxygen species mediated mtDNA damage can occur directly at the sugar-phosphate backbone, at the bases, or in the form of single and double strand breaks ^20^. Unfortunately, most of the antioxidant-supplementation regimens do not increase longevity, as predicted by the free radical theory of aging. Intracellular ROS are generated in multiple compartments and by multiple pathways. Important contributors in this case are NADPH oxidases, cyclooxygenases, and lipid metabolism enzymes ^21^. Despite several non-mitochondrial contributors, almost 90% of cellular ROS are still generated in mitochondria. In some cases, long-lived species were not only found to produce less ROS but also showed less oxidative damage ^22^. Similarly, various animal and human studies suggest that the decline in muscle mitochondria is a leading factor for muscular abnormalities ^23^.

Aged monkeys showed enhanced DNA damage and reduced transcription of mtDNA compared to young ones ^24^. D-gal-induced aging rats are important animal model of aging, and the level of mtDNA deletions was found to be significantly more in the hippocampus of D-gal-treated rats compared to controls ^25^. NADPH oxidase (NOX) generates ROS while transporting electrons across the mitochondrial membrane. Similarly, uncoupling protein 2 (UCP2) transports anions and protons across the mitochondrial membrane, and also controls ROS generation. In case of D-gal-induced animal model of aging, damaged mitochondrial ultrastructure was seen in the hippocampus region along with increased production of NOX and UCP2. Nicotinamide adenine dinucleotide (NAD+) is a key electron transporter in mitochondria. NAD+ depletion may play a prominent role in the aging process, not only by limiting energy production, but also by compromising DNA repair and genomic signaling as NAD+ is an important substrate for the nuclear repair enzymes ^21^. Poly(ADP-ribose) polymerase (PARP) controls inflammatory immune responses, and hyperactivation of PARP-1 is known to activate mitochondrial pathway of apoptosis ^26^. Age-associated increase in oxidative nuclear damage was found to be associated with PARP-induced NAD+ depletion and absence of SIRT1 activity in rodents ^26^. Ercc1 mutant mice, which are deficient in DNA repair pathways, show accelerated aging and progressive memory loss ^27^. Defective oxidative phosphorylation, mutated mtDNA, or mitochondrial ROS have also been documented in cases of tumorigenesis ^28^. Oxidative stress in the cardiovascular system is known to cause accumulation of reactive oxygen and nitrogen species, which increase leukocyte adhesion and endothelial permeability ^29^. NFκB is one of the most important transcription factor that is known to be involved in important signaling pathways, development, and several diseases. Hypoxia-Inducible Factor (HIF-1) is a protein that not only protects from hypoxia-induced damage, but is also important for smooth functioning of immune system and key metabolic pathways. In an interesting study, ROS, NFκB- and HIF1-activation in the tumor microenvironment induced accelerated aging in rodents, which subsequently caused stromal inflammation and altered cancer cell metabolism ^30^. Certain dietary treatments or enrichment of mitochondrial membranes with oxidant-resistant fatty acids were found to increase life span in rodents ^31^. Monounsaturated-fatty-acid-rich diet prevented the accelerated mtDNA mutations in the brain mitochondria from aged animals. Therefore, changes in mtDNA that gradually accumulate in a variety of tissues during aging appear to be involved in onset of various diseases ^32^ and a better understanding of mitochondrial biology is required in this perspective. mtDNA ligase is essential for cell survival particularly because of its role in base excision repair pathway ^33^.

## Conclusions

Mitochondria are of central importance in eukaryotic cells. However, mtDNA is more prone to damage, and mtDNA repair pathways are inadequate. Together, these problems might frequently lead to unrepaired mtDNA lesions, and defective energy metabolism. mtDNA damage has been frequently shown to be involved in initiation and progression of several diseases including various types of neurodegenerative disorders, cancer, stroke, heart-diseases, and diabetes. There is an urgent need for detailed investigation in this area, to find out the mitochondrial contribution to various diseases, so that improved prevention measures and cures can be developed.
