# Supplementary material for: Mitochondrial DNA Damage and Diseases
Source: F1000Res. 2015 Jul 1;4:176. [Version 1] doi: 10.12688/f1000research.6665.1 (PMC4962287; doi:10.12688/f1000research.6665.1)
Supplement: Peer review history

# Peer review: Referee response for version 1

**DOI:** 10.5256/f1000research.7161.r14538

**Published:** 2016-07-26

**Authors:** Ullas Kolthur-Seetharam

**Affiliations:** 1 Department of Biological Sciences, Tata Institute of Fundamental Research (TIFR), Mumbai, Maharashtra, India

The review titled “Mitochondrial damage and diseases” has attempted to give an overview of the current literature on the causes of mtDNA damage and its effect in disease and aging. Although the authors seem to have cited a lot of literature in this regard, they have not been able to present their views in a collected manner.

 The language used is extremely colloquial (e.g. Page 2 “Furthermore, the size of mtDNA is very small (16.6 kb in humans), and mitochondrial codon-usage is also different” and “Reactive oxygen species (ROS) are very reactive oxygen-containing molecules”) and disjointed. In the last paragraph, it is not clear why the authors are suddenly talking about nuclear DNA damage as the review concentrates on mtDNA damage.

 The authors move from one point to another without clarity of thought and without drawing relevant conclusions supported by multiple references. References are missing in multiple places. There also seem to be some cases of mis-referencing.

 The authors could discuss a little more in their introduction about the DNA repair pathways that do exist in the mitochondria. From their text, it seems that any damage induced in the mitochondria is never repaired. The authors should also discuss the current techniques that are available for rectifying mtDNA damage like genome editing. This is particularly important as they stress this in their conclusion. Also, they should speculate on the effect of DNA damage, NAD loss, and accelerated aging based on previous literature.

I have read this submission. I believe that I have an appropriate level of expertise to confirm that it is of an acceptable scientific standard, however I have significant reservations, as outlined above.

# Peer review: Referee response for version 1

**DOI:** 10.5256/f1000research.7161.r14777

**Published:** 2016-07-05

**Authors:** George Shinomol

**Affiliations:** 1 Department of Neurochemistry, Neurotoxicology laboratory, Neurobiology Research Centre (NRC), National Institute of Mental Health and Neuroscience (NIMHANS), Bangalore, Karnataka, India

The article (Review) titled "Mitochondrial DNA damage and diseases" is a good work. However there are certain shortcomings that the authors need to address.

 In general there is much refinement needed for language

1. The abstract lacks clarity and it doesn’t actually represent the entire article. It needs to be re written
2. The paper is unorganised .The consequences of mitochondrial damage could have been described under various appropriate subheadings, e.g.: Neurodegenrative diseases, Aging, Cardiovascular diseases etc. This could have increased the clarity and quality of this review
3. There also could have been mentioned some details on amelioration of the effects using
  mitochondria targeted therapies
4. Include recent references

I have read this submission. I believe that I have an appropriate level of expertise to confirm that it is of an acceptable scientific standard, however I have significant reservations, as outlined above.

# Peer review: Referee response for version 1

**DOI:** 10.5256/f1000research.7161.r11088

**Published:** 2015-12-07

**Authors:** Linda Bergersen

**Affiliations:** 1 Department of Oral Biology, University of Oslo, Oslo, Norway

This short review by Gyanesh Singh​ *et al*. provides useful and up-to-date information on the roles of mtDNA in disease. 

Abnormalities in mtDNA may affect all organs of the body, but cause symptoms primarily in tissues that are dependent on high energy production. Deficient mtDNA maintenance contributes to conditions as diverse as normal aging, neurodegenerative disease, diabetes, cardiovascular disease, and cancer.

The authors should give more precise and explicit reference to the repeated assertion that "mtDNA repair pathways are inadequate" (e.g. in the Abstract and Conclusions sections), or moderate these statements. The statement reads as indicating that even when the mtDNA repair mechanisms function normally, they are inadequate. Is there direct evidence for this?

On p2, the authors correctly point out that it is difficult to distinguish the effects of genotoxic agents on mtDNA, "as agents that damage mtDNA also damage nuclear DNA". The authors should reference work on transgenic animal models with damage specifically in mtDNA (e.g., Trifunovic A et al 2004 Nature; Lewis W et al 2007 Lab Invest; Lauritzen KH et al 2010 Mol Cell Biol​).

Typography: in "NAD+" the "+" should be corrected to superscript.

Please check the text for grammatical errors, e.g., on p2 "...the only DNA polymerase known to be present in the mitochondria, have

low frameshift fidelity, and, is believed to...": "have" should be corrected to "has".

I have read this submission. I believe that I have an appropriate level of expertise to confirm that it is of an acceptable scientific standard.
